# Retrosternal parathyromatosis in a patient with prior total parathyroidectomy

**DOI:** 10.1093/jscr/rjad256

**Published:** 2023-06-05

**Authors:** Tariq Saleh, Marwan Alaswad, Abdullah Otry, Waleed Saleh

**Affiliations:** College of Medicine, Alfaisal University, Riyadh, Saudi Arabia; College of Medicine, Alfaisal University, Riyadh, Saudi Arabia; College of Medicine, Alfaisal University, Riyadh, Saudi Arabia; Department of Thoracic Surgery, King Faisal Specialist Hospital and Research Center, Riyadh, Saudi Arabia

## Abstract

Parathyromatosis is a rare cause of recurrent primary hyperparathyroidism that often follows surgical removal of the parathyroid gland. Foci of parathyromatosis are most commonly found in the neck, mediastinum, and sites of autotransplantation. A 36-year-old male with renal failure and prior parathyroidectomy presented with generalized bone pain, for which laboratory investigations revealed hyperparathyroidism. Preoperative coil localization was utilized followed by thoracoscopy using fluoroscopy for resection of ectopic parathyroid tissue. The specimen was sent to histopathology, which revealed multiple nodules of hypercellular parathyroid tissue, consistent with the diagnosis of parathyromatosis. Parathyromatosis is a rare cause of recurrent hyperparathyroidism, with surgical removal being the only curative option. Follow-up is essential as it can commonly recur.

## INTRODUCTION

Parathyromatosis is an extremely uncommon cause of persistent, recurrent primary hyperparathyroidism, initially described by Palmer *et al*. [1]. Multiple theories have been proposed regarding its development; however, it usually occurs following surgical removal of the parathyroid glands [2]. Parathyromatosis is seen mostly in patients with renal failure, and foci of parathyromatosis are usually located in the mediastinum and neck as reported by previous case studies [3, 4]. We report a case of a 36-year-old male with ectopic parathyroid tissue in the upper retrosternal region localized by preoperative coil technique that was diagnosed with parathyromatosis.

## CASE PRESENTATION

**Figure 1:**
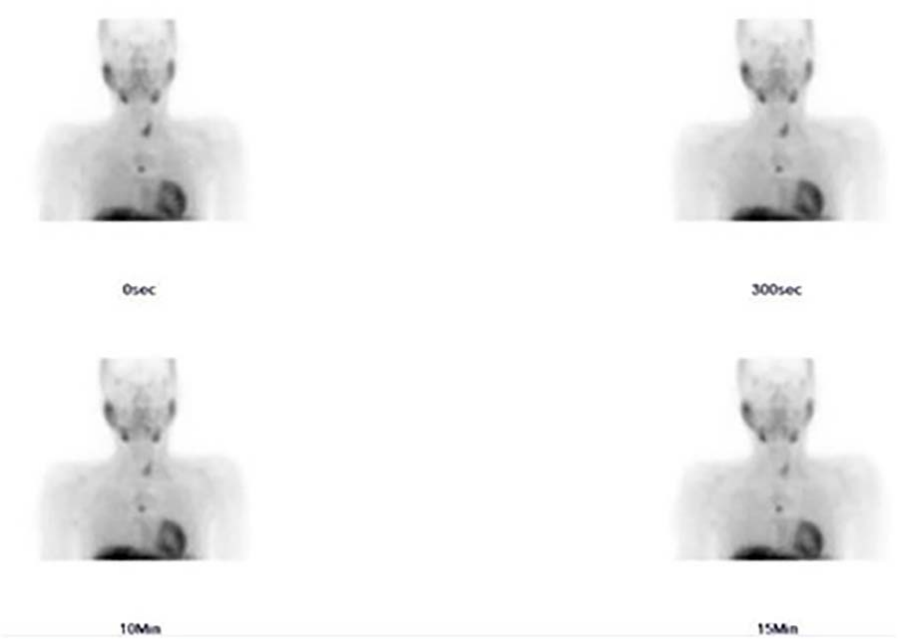
Sestamibi scan: hyperactive parathyroid tissue in the retrosternal area.

**Figure 2:**
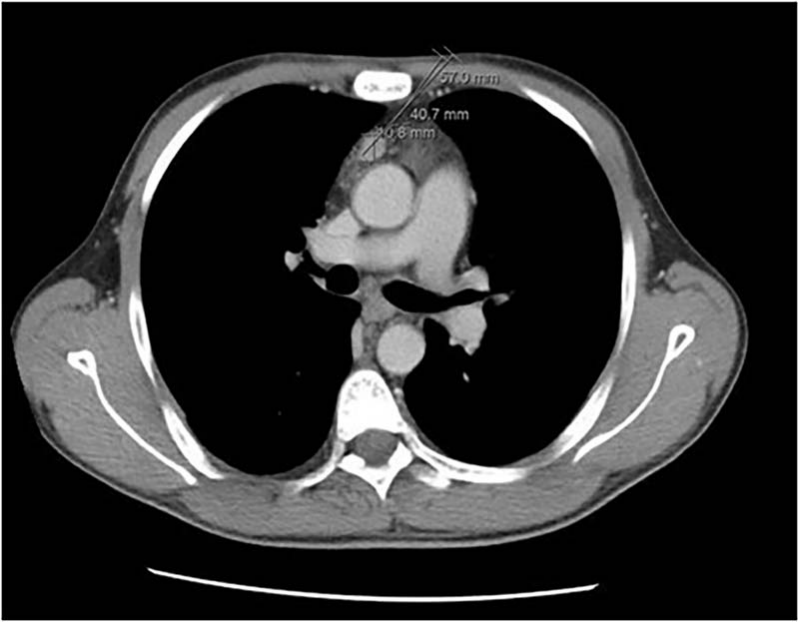
Chest CT revealing a retrosternal lesion measuring 57 mm anterior to the ascending aorta.

**Figure 3:**
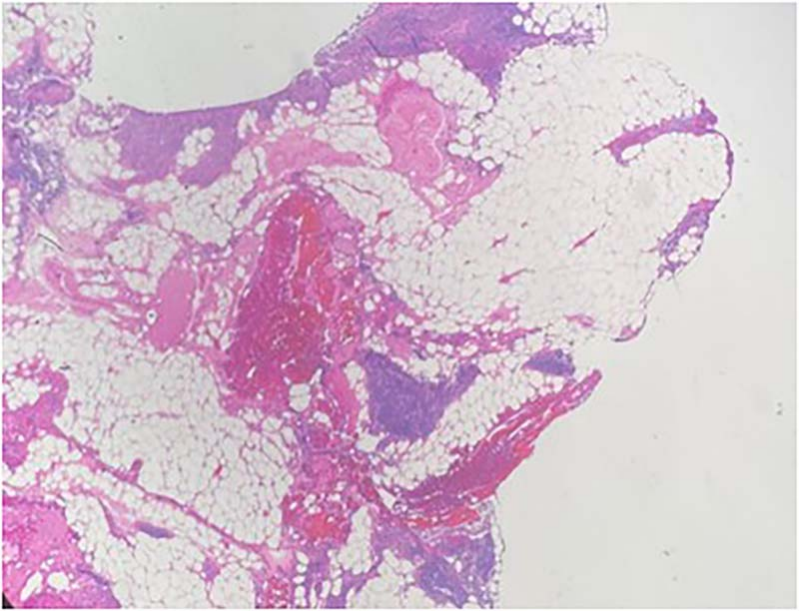
Multiple nodules of hypercellular parathyroid tissue within unremarkable thymic tissue.

**Figure 4:**
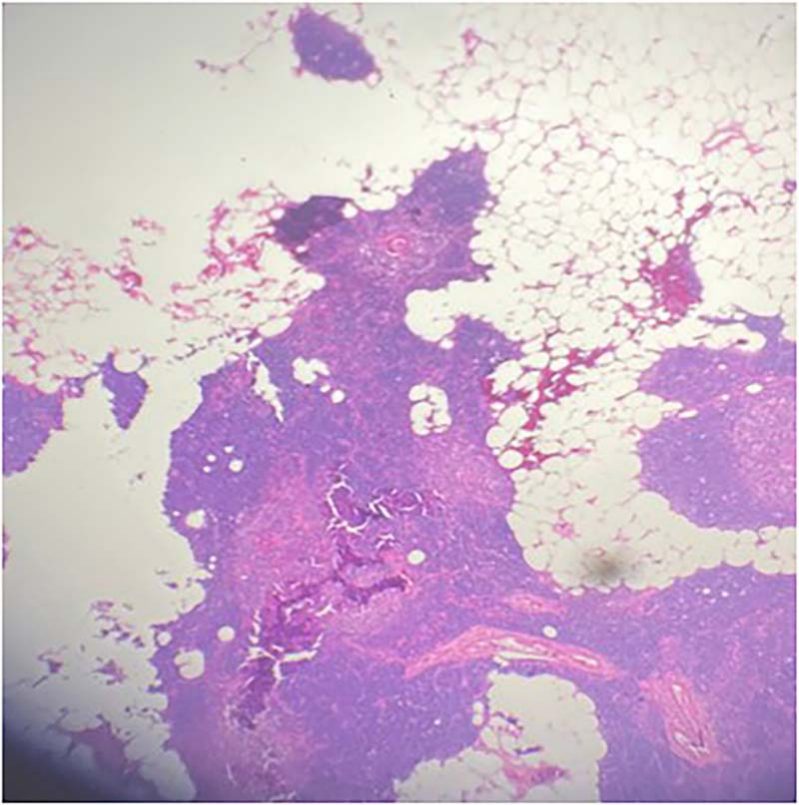
Multiple nodules of hypercellular parathyroid tissue within unremarkable thymic tissue.

A 36-year-old male with a history of end-stage renal disease (because of polycystic kidney disease) on dialysis, for which he is on paricalcitol and sevelamer, and total parathyroidectomy with autotransplantation in the left forearm performed in 2011 for secondary hyperparathyroidism presented with generalized bone pain. Heart rate was 114 bpm, respiratory rate was 18 breaths/min, oxygen saturation was 96% and blood pressure was 136/72 mmHg. Laboratory investigations were ordered and revealed high PTH (>1300 pg per ml), low calcium (2.7 mg per dl) and low phosphate (1.8 mg per dl). A sestamibi scan was ordered and revealed hyperactive parathyroid tissue in the upper retrosternal area (Fig. 1). Removal of the ectopic parathyroid tissue was planned; however, the lesion was not localizable intraoperatively, and further chest CT with preoperative coil localization was performed, which revealed a retrosternal ectopic parathyroid tissue anterior to the ascending aorta (Fig. 2).Right thoracoscopy using fluoroscopy for ectopic parathyroid removal and lymph node biopsy was performed, and the specimens were sent to histopathology, which showed multiple nodules of parathyroid tissue with benign lymph nodes (Figs 3 and 4). A final diagnosis of parathyromatosis was made. The patient was discharged in good condition without complaints of bone pain. Upon 2-week follow-up, the patient was symptom-free and laboratory investigations revealed phosphate of 2.1 mg per dl, calcium of 2.4 mg per dl and an elevated PTH of 350 pg per ml, for which a sestamibi scan was performed and showed no evidence of uptake in the neck and mediastinum but revealed uptake in the left forearm from the previous autotransplantation.

## DISCUSSION

Parathyromatosis, originally described by Palmer *et al*. [1], is an extremely rare cause of recurrent primary hyperparathyroidism, with only 20 reported cases in the English literature from 1975 to September 2018 [2]. Three theories have been proposed regarding the development of parathyromatosis: low-grade parathyroid malignancy, seeding and secondary implantation into the surrounding tissues of the parathyroid gland during parathyroidectomy and activation of the embryological remnants of the parathyroid gland [5]. It is important to note that parathyromatosis is more common in patients with end-stage renal disease, as is the case with our patient [4]. Parathyromatosis foci are most frequently localized in the neck and sites of parathyroid gland autotransplantation such as the forearm or sternocleidomastoid muscle [2]. Foci of parathyromatosis have also been described in subcutaneous adipose tissue, adjacent to the recurrent laryngeal nerve, retrosternal area, carotid sheath, thymus, upper mediastinum and tracheoesophageal groove [5].

The most important disease to be excluded from the differential diagnosis of parathyromatosis is parathyroid cancer [5]. Patients with parathyroid cancer have more profound hypercalcemia and metastatic disease compared with those with parathyromatosis [5]. Moreover, parathyromatosis presents as small and multiple nodules intraoperatively, whereas parathyroid cancer is usually a solitary tumor [3].

Preoperative diagnostic modalities used for parathyromatosis include ultrasound and technetium Tc 99 m sestamibi scan [4, 6]. Other options include 4D-computed tomography [7]. To the best of our knowledge, we report the first case of preoperative coil localization followed by thoracoscopy using fluoroscopy in the management of parathyromatosis foci. Intraoperatively, parathyromatosis is frequently surrounded by adherent fibrous tissue because of prior surgery, which may resemble parathyroid cancer [3].

The mainstay treatment of parathyromatosis is en bloc resection of all foci [3]. However, the outcome and effectiveness of the surgery are variable because of the difficulty of intraoperative detection of preoperatively non-observable foci [4]. We believe our approach of preoperative coil localization followed by thoracoscopy using fluoroscopy improves the outcome of surgery and reduces operative time. Multiple surgeries may be required to achieve complete remission because recurrence may occur at different silent foci [2, 8]. Medical management of parathyromatosis includes a calcimimetic agent along with an active vitamin D analog, with Daphnis *et al*. [9] reporting success using cinacalcet and paricalcitol.

## CONCLUSION

Parathyromatosis is an extremely rare cause of recurrent hyperparathyroidism. Parathyroid cancer is the main differential diagnosis that must be excluded. The only curative option is complete surgical removal of all foci, which requires adequate preoperative localization studies. In our case, we implemented preoperative coil localization followed by thoracoscopy using fluoroscopy, which we believe eliminates the failure rate of surgery and shortens the operative time. Postoperatively, patients should be monitored closely with laboratory and radiological screening, since parathyromatosis may reoccur during the follow-up period.

## CONFLICT OF INTEREST STATEMENT

None declared.

## FUNDING

None.

## DATA AVAILABILITY

All data underlying the results are available as part of the article and no additional source data are required.
